# Royal Canadian Mounted Police cadets’ exposure to potentially psychologically traumatic events during the Cadet Training Program

**DOI:** 10.1002/jts.23115

**Published:** 2024-12-20

**Authors:** Katie L. Andrews, Kirby Q. Maguire, Laleh Jamshidi, Tracie O. Afifi, Jolan Nisbet, Robyn E. Shields, Taylor A. Teckchandani, Gordon J. G. Asmundson, Alain Brunet, Lisa M. Lix, Shannon Sauer‐Zavala, Jitender Sareen, Terence M. Keane, J. Patrick Neary, R. Nicholas Carleton

**Affiliations:** ^1^ Department of Psychology, Faculty of Arts University of Regina Regina Saskatchewan Canada; ^2^ Zilber College of Public Health University of Wisconsin–Milwaukee Milwaukee Wisconsin USA; ^3^ Department of Community Health Sciences University of Manitoba Winnipeg Manitoba Canada; ^4^ Department of Politics and International Studies University of Regina Regina Saskatchewan Canada; ^5^ Anxiety and Illness Behaviours Laboratory Department of Psychology University of Regina Regina Saskatchewan Canada; ^6^ Department of Psychiatry McGill University Montreal Quebec Canada; ^7^ Douglas Institute Research Center Montreal Quebec Canada; ^8^ Treatment Innovation for Psychological Services Research Program, Department of Psychology University of Kentucky Lexington Kentucky USA; ^9^ Department of Psychiatry University of Manitoba Winnipeg Manitoba Canada; ^10^ Behavioral Science Division, National Center for PTSD VA Boston Healthcare System & Boston University Chobanian & Avedisian School of Medicine Boston Massachusetts USA; ^11^ Faculty of Kinesiology and Health Studies University of Regina Regina Saskatchewan Canada

## Abstract

Lifetime exposures to potentially psychologically traumatic events (PPTEs) among Royal Canadian Mounted Police (RCMP) cadets starting the Cadet Training Program (CTP) appear lower than exposures reported by serving RCMP, but the prevalence of PPTE exposures during the CTP remains unknown. The current study assessed PPTE exposures during the CTP and examined associations with mental disorders among RCMP cadets. Participants were cadets (*n* = 449, 24.7% women) from the larger RCMP Longitudinal Study who self‐reported critical incidents, PPTE exposures, and mental health disorder symptoms at pretraining and predeployment. Most participants reported no exposures to a PPTE (*n* = 374, 83.3%) during the CTP. Participants who reported any PPTE exposure (*n* = 75, 16.7%; i.e., direct or indirect) most commonly reported serious transport accidents, physical assault, and sudden accidental death. The most common direct PPTEs (i.e., “happened to me”) during the CTP were physical assault (*n =* 13), other unwanted or uncomfortable sexual experience (*n* = 11), and serious transportation accident (*n* = 8). The total number of PPTE types reported at predeployment was associated with increased odds of screening positive for any mental health disorder, a*OR* = 1.22, 95% CI [1.01, 1.49], *p* = .049, and positively associated with mental health disorder symptoms, *p*s < .001. These results provide the first assessment of PPTE exposure among RCMP cadets during the CTP, indicating that 16.7% of cadets experience PPTEs directly or indirectly. The PPTEs reported by cadets may help inform additional opportunities to further increase safety during training.

Royal Canadian Mounted Police (RCMP) evidence a high prevalence of exposure to potentially psychologically traumatic events (PPTEs) as well as a high prevalence of mental health disorders (Carleton et al., 2019, 2018). Longstanding notions suggest that mental health challenges among RCMP members are the result of lifetime PPTE exposures (i.e., prior to service) and preexisting mental health concerns. Recent evidence indicates that new RCMP cadets starting the Cadet Training Program (CTP) report fewer PPTE exposures than serving RCMP members (Andrews et al., 2023) and better mental health than both serving RCMP members and the general public (Carleton et al., 2023). Therefore, the high prevalence of PPTE exposure and mental health disorders among serving RCMP members are likely the result of service experiences and not preexisting PPTE exposure histories and mental health challenges. The current evidence indicates that as individuals progress through their RCMP careers as active duty officers, PPTE exposures likely increase, iteratively increasing the risk of mental health disorders.

Previous evidence suggests that exposure to PPTEs can begin to accumulate within the first 12 months of police service, leading to increased symptoms of posttraumatic stress disorder (PTSD; Hodgins et al., 2001; Huddleston et al., 2006). The extant research has assessed PPTE exposure starting from recruit training and spanning the first 12 months of active duty service, which potentially conflates exposure to PPTEs experienced before or during training with exposure experienced through subsequent active duty service. No known studies have examined PPTE exposures during recruit training and the associated impacts of such exposure on mental health. Recruit training is responsible for preparing new hires for the challenging career of policing. To do so, most police training programs implement a stress‐based training method involving both psychological and physical demands (Reaves, 2016). Thus, exposure to various PPTEs may begin to accumulate as early as recruit training. Recruits in the training environment may also be vulnerable to specific PPTEs that may increase the risk of developing mental health challenges during training and onward into their careers. Clarifications about the prevalence of PPTE exposures (both overall and to different PPTE types) and associated mental health challenges during training might inform recruitment, selection, training, and early intervention practices for members in active duty service.

The RCMP Longitudinal PTSD Study (i.e., The RCMP Study) was designed to assess a multimodal mental health solution that includes evidence‐based biopsychosocial assessments and evidence‐informed integrated cadet mental health training before and after training and annually for 5 years (Carleton et al., 2022). The RCMP Study provides the opportunity to examine the frequency and diversity of exposure to PPTEs exposure experienced by cadets both during the CTP and prior to deployment as active duty RCMP officers. The RCMP CTP is a rigorous 26‐week program at the RCMP Depot in Regina, Saskatchewan (Canada). During the CTP, cadets study and practice well beyond 8 hr of scheduled classroom time per day (Hembroff & Kratzig, 2020), with training including applied police sciences, firearms, police defensive tactics, driving, simulation training, operational conditioning, and drill and deportment (RCMP, 2019). Understanding the experiences of cadets during the CTP and prior to deployment can inform recruitment and retention by providing insight into aspects of the training experience that may impact cadets’ mental health and identifying resources needed to further support cadets during training.

Previous research from the RCMP Study has focused on examining cadets’ lifetime PPTE exposure to help identify factors related to mental health risk and resilience among recruits prior to training (Andrews et al., 2023). The current study provides estimates of RCMP cadets’ PPTE exposures between pretraining and predeployment, which encompasses the 26‐week CTP. The current paper was designed to (a) assess the prevalence of PPTE exposures experienced by RCMP cadets during the CTP, (b) compare PPTE exposures during the CTP by sociodemographic characteristics, and (c) test for associations between PPTE exposures (i.e., number and type) and screening positive for diverse mental health disorders. We hypothesized that PPTE exposures during the CTP would be associated with mental health disorder symptoms and subsequent positive screens.

## METHOD

### Participants and procedure

Full details on the RCMP Study methods are available in the published protocol paper (Carleton et al., 2022). After obtaining informed consent, data were collected using an online self‐report survey available in English or French through Qualtrics. The RCMP Study was approved by the University of Regina Institutional Research Ethics Board (File No. 2019–055) and the RCMP Research Ethics Board (File No. SKM_C30818021312580). The RCMP Study was also approved through a Privacy Impact Assessment as part of the overall National Administrative Records Management System approval (201611123286) and Public Services and Procurement Canada approval (201701491/M7594174191). Data were collected from April 2019 to March 2022. Data collection was paused due to COVID‐19 restrictions closing the RCMP Depot from December 2019 to March 2020. The current paper focuses on longitudinal data collected at pretraining and predeployment, which included self‐reported PPTE exposures and mental health disorder symptoms.

Participants were RCMP cadets participating in the RCMP Study who completed the 26‐week CTP. To qualify for the CTP, the RCMP requires cadets to be Canadian citizens or permanent residents; be 19–57 years old; be able to fluently read, write, and speak either English or French (Hembroff & Kratzig, 2020); and meet several recruiting requirements (i.e., security clearances, medical examinations, a polygraph test, and minimum physical standards). Any individuals who qualified for the CTP could participate in the RCMP Study. At pretraining, a total of 776 cadets completed the survey. The sample lost a total of 323 participants to attrition from pretraining to predeployment. The current study includes data from RCMP cadets (*n =* 449) who completed the survey at pretraining and predeployment, herein referred to as “completers.” As presented in Supplementary Table 1, most participants were male (73.9%), identifying as men (74.2%), 19–29 years old (62.8%), White (77.1%), single (45.9%), and from Western Canada (53.9%; i.e., British Columbia, Alberta, Saskatchewan, and Manitoba). The majority of participants had completed some postsecondary education (43.9%) and had no previous public safety or military experience (61.2%).

### Measures

#### Critical incident exposure

At pretraining, participants reported lifetime critical incident exposure, and at predeployment, participants reported critical incident exposure that had occurred since the last time they completed the questionnaire (i.e., during the CTP). Critical incidents included: witnessed line‐of‐duty deaths; experienced or witnessed serious line‐of‐duty injuries; disasters or multicasualty incidents; incidents involving the unusual or sudden death of children or harm of children; incidents that seriously threatened [the respondent's] life or the life of a colleague; incidents for which the victims were relatives or friends; the suicide of a close colleague or a superior; sexual harassment, assault, or discrimination; or any other PPTE. Additional response options allowed participants to indicate “prefer not to answer” or “none.” To protect participant anonymity due to small sample sizes across critical incident categories, critical incident categories were collapsed to “any critical incident exposure” (e.g., exposure to one or more critical incidents).

#### Potentially psychologically traumatic event exposure

The extended version of the Life Events Checklist for *DSM‐5* (LEC‐5; Weathers, Blake, et al., 2013) was used to assess participant PPTE exposure per PTSD Criterion A as outlined in the *Diagnostic and Statistical Manual of Mental Disorders* (5th ed.; *DSM‐5*; American Psychiatric Association [APA], 2013). At pretraining, participants were asked to report lifetime PPTE exposure, and at predeployment, they were asked to report PPTE exposure they had experienced since they had last filled out the questionnaire. For each exposure type, response options are “happened to me,” “witnessed it,” learned about it,ʼʼ and “part of my job.” Participants reported on the exposure modality (e.g., indirect or direct), and all endorsed experiences were treated as an exposure. For the current study, the LEC‐5 was modified to ask participants to report the number of times they were exposed to each endorsed PPTE type. The total number of PPTE types experienced was quantified by summing exposure frequencies across the 17 items. If a participant reported exposure to more than one PPTE type, they were also asked to select the PPTE they considered the “worst” or to be currently causing them the most distress. Due to a technical error in the survey at predeployment, frequencies and worst PPTEs could not be reported in the current study, which has been noted as a limitation.

**FIGURE 1:**
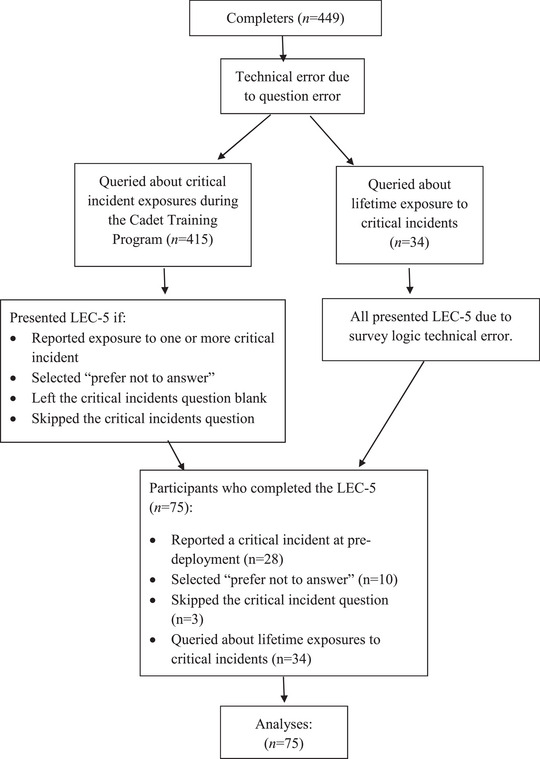
Data collection technical errors flow diagram. *Note*: LEC‐5 = Life Events Checklist for *DSM‐5*.

Technical errors related to the critical incident questions resulted in some adjustments to the data (see Figure 1). At pretraining, all participants (*n* = 449) completed the critical incident questionnaire and the LEC‐5. At predeployment, most participants (*n* = 415) were queried about critical incident exposure that had occurred since they last completed this questionnaire and were presented with the LEC‐5 if they reported exposure to one or more of the critical incidents described previously (*n* = 28) or selected “prefer not to answer” (*n* = 10). Participants who left the critical incident selection blank or skipped the question (*n* = 3) did not trigger the survey logic functions and, thus, also received the LEC‐5. A subset of participants (*n* = 34) mistakenly received a query for lifetime exposure to critical incidents; this subset of participants also received the LEC‐5 due to a technical error with the survey logic functions. Therefore, a total of 75 (16.7%) participants completed the LEC‐5.

#### PTSD

The 20‐item PTSD Checklist for *DSM‐5* (PCL‐5; Weathers, Litz, et al., 2013) was used to assess PTSD symptoms in the past month. Participants were asked to rate each item on a scale of 0 (*not at all*) to 4 (*extremely*), with higher scores indicating higher symptom levels. For the PCL‐5, a positive screen required participants to report exposure to at least one LEC‐5 item, meet the minimum *DSM‐5* criteria (APA, 2013) for each PTSD symptom cluster subscale (e.g., intrusions, avoidance, negative alterations in cognitions and mood, and alterations in arousal and reactivity), and exceed the clinical cutoff score of 32 or higher (Weathers, Litz, et al., 2013). The PCL‐5 has demonstrated strong internal consistency (Cronbach's α = .94) and good test–retest reliability (*r* = .82) in populations exposed to PPTEs (Blevins et al., 2015). In the current sample, internal consistency was very good, Cronbach's alpha was .93.

#### Major depressive disorder

The nine‐item Patient Health Questionnaire (PHQ‐9; Kroenke et al., 2001) was used to assess symptoms of major depressive disorder (MDD) symptoms experienced in the past 2 weeks. Participants were asked to rate symptoms on a scale of 0 (*not at all*) to 3 (*nearly every day*), with higher scores indicating more severe depressive symptoms. For the PHQ‐9, a positive screen required a score of 9 or higher (Manea et al., 2015). The PHQ‐9 has demonstrated good internal consistency (Cronbach's α = .89) and test–retest reliability (*r* = .84) in the general population (Kroenke et al., 2001). In the present sample, internal consistency was good, Cronbach's alpha = .83.

#### Panic disorder

The Panic Disorder Symptoms Severity Scale–Self‐Report (PDSS‐SR; Shear et al., 1997) was used to assess panic disorder (PD) symptoms related to a panic attack in the past week rated on a 5‐point Likert scale ranging from 0 (*none*) to 4 (*extreme*). A positive screen required the PDSS‐SR total score to be greater than 7. The PDSS‐SR has demonstrated a strong internal consistency (Cronbach's α = .92) and an intraclass correlation coefficient of .81 (Houck et al., 2002). In the current sample, internal consistency was adequate, Cronbach's α = .70.

#### Generalized anxiety disorder

The seven‐item Generalized Anxiety Disorder scale (GAD‐7; Spitzer et al., 2006) was used to assess symptoms of generalized anxiety disorder (GAD) in the previous 2 weeks. Participants were asked to rate each item on a scale of 0 (*not at all*) to 3 (*nearly every day*), with higher scores indicating more severe symptoms. A positive screen required a GAD‐7 total score of 9 or higher (Spitzer et al., 2006). The GAD‐7 has shown good reliability; construct, criterion, procedural, and factorial validity (Spitzer et al., 2006); and good internal consistency (Cronbach's α = .89), with interitem correlations of .45–.65 in a community sample (Löwe et al., 2008). In the current sample, internal consistency was good, Cronbach's α = .89.

#### Social anxiety disorder

The 14‐item Social Interaction Phobia Scale (SIPS; Carleton et al., 2009) was used to assess for social anxiety disorder (SAD) symptoms rated on a 5‐point Likert scale ranging from 0 (*not at all characteristic of me*) to 4 (*entirely characteristic of me*). A positive screen for SAD requires a SIPS total score to be greater than 20 (Carleton et al., 2009). The SIPS has demonstrated overall excellent internal consistency (Cronbach's α = .92) and convergent and discriminant validity in a large and independent sample (Reilly et al., 2012). In the current sample, internal consistency was good, Cronbach's α = .88.

#### Alcohol use disorder

The 10‐item Alcohol Use Disorders Identification Test (AUDIT; Barbor et al., 1989) was used to assess symptoms of alcohol use disorder (AUD), with Likert scale responses that vary across items. A positive screen for AUD required the total AUDIT score to be greater than 15 (Gache et al., 2005). The AUDIT has demonstrated good internal consistency (Cronbach's α = .81) and good test–retest reliability (*r*s = .83–.95) in the general population and good internal consistency in a police‐specific population (Cronbach's α = .81; Daeppen et al., 2000; Davey et al., 2000; Reinert & Allen, 2007). In the current sample, internal consistency was adequate, Cronbach's α = .68.

### Data analysis

Participants were categorized based on their responses to the critical incident question at pretraining and predeployment. Prevalence of exposure to any critical incident (e.g., exposure to at least one or more critical incidents and, by extension, one or more PPTE) was categorized relative to “prefer not to answer” at pretraining; participants did not have the option to select “none” at pretraining. Prevalence of exposure to any critical incident was grouped relative to “prefer not to answer” and no critical incidents and, by extension, no PPTE exposure during the CTP based on predeployment responses.

The frequencies and percentages of sociodemographic characteristics (i.e., sex, gender, age group, ethnicity, marital status, province of residence, level of education, and previous public safety or military experience) were calculated for comparisons. Means and standard deviations for the total number of endorsed PPTE types were calculated for lifetime exposure at pretraining and exposure during the CTP at predeployment for completers. A series of *t* tests and one‐way analysis of variance (ANOVA) *F* tests were conducted to examine differences in the mean total number of PPTE types endorsed across sociodemographic characteristics at pretraining and predeployment. Nonresponse bias analyses were conducted using independent‐samples *t* tests and independent‐samples proportion tests to test for main effects between completers (*n* = 449) and noncompleters (*n* = 323) at predeployment. All tests were two‐sided and set at an alpha level of .05. Holm–Bonferroni adjustments were applied to alpha levels in post hoc analyses to control the familywise Type I error rate in multiple comparisons (Holm, 1979). Cohen's *d* effect sizes were utilized in *t* tests, with values of 0.20, 0.50, and 0.80 indicating small, medium, and large effects, respectively (Cohen, 2013). Partial eta squared (η_p_^2^) was reported as a measure of effect size for ANOVA tests, assessing the proportion of variance explained by a particular variable while accounting for the variance explained by other variables in the model; partial eta squared values of .01, .06, and .14 indicate small, medium, and large effects, respectively. Missing data were addressed using listwise deletion for all analyses, resulting in complete case analysis. No cases were omitted due to partially incomplete records, and, therefore, all missing data were due to study attrition. Exposure prevalence was calculated as the endorsement of any exposure for lifetime PPTEs (i.e., ever exposure) at pretraining and exposure during the CTP at predeployment, then by exposure modality (i.e., direct vs. indirect per LEC‐5 response). Direct exposure (i.e., “happened to me”) prevalence for each PPTE type was also examined across gender categories at predeployment.

Multivariable logistic regression models were conducted to test the associations between positive screens for any mental health disorders (e.g., at least one or more positive screens for PTSD, MDD, GAD, SAD, PD, or AUD) as the dependent variable and the total number of different PPTE types experienced and ever‐exposure to any PPTE type at predeployment as independent variables. Simultaneous linear regressions were conducted to assess the associations between the total number of PPTE types experienced and mental health disorder symptom scores at predeployment.

All logistic regression models were adjusted for sociodemographic covariates (e.g., sex, gender, age, marital status, ethnicity, province of residence, level of education). Adjusted odds ratios (a*OR*s) and 95% confidence intervals (CI) were estimated. Concordance statistics (C‐statistics) were also calculated as a measure of the predictive performance of the logistic regression models (Austin & Steyerberg, 2012). The obtained C‐statistics (range: .829–.903) were strong, indicating the models correctly classified the outcomes (i.e., positive screens for any mental health disorders). Post hoc sensitivity analyses were conducted for all logistic regression models to assess differences in model fit and the observed power associated with the inclusion of sociodemographic covariates.

## RESULTS

At pretraining, many participants (*n =* 201) reported exposure to at least one critical incident, but some preferred not to answer (*n =* 85). At pretraining, all participants received the LEC‐5 and reported lifetime PPTE exposure. At predeployment, most participants reported exposure to no critical incidents (*n* = 374, 83.3%) and, by extension, no PPTEs, during the CTP; relatively few participants reported any critical incident exposure (*n* = 28, 6.7%) during the CTP. At predeployment, 16.7% (n = 75) of participants received the LEC‐5 and reported PPTE exposures during the CTP.

Supplementary Table S1 presents the differences in the total number of PPTE types experienced across sociodemographic characteristic categories at pretraining and predeployment. At pretraining, completers who had previous public safety or military experience reported exposure to a statistically significantly higher total number of different PPTE types than those with no previous experience, *t*(406) = 4.90, *d* = 0.516, *p* < .001. At pretraining, there were no statistically significant differences in the mean total number of different PPTE types endorsed based on sex *d* = 0.03; gender, *d* = 0.004; age, η_p_^2^ = .01; marital status, η_p_^2^ = .006; ethnicity, η_p_^2^ = .006; or province of residence, η_p_^2^ = .012. At predeployment, there were no statistically significant differences in the mean total number of PPTE types endorsed based on sex, *d* = 0.081; gender, *d* = 0.002; age, η_p_^2^ = .000; ethnicity, η_p_^2^ = .010; marital status, η_p_^2^ = .018; province of residence, η_p_^2^ = .010; or previous public safety or military experience, *d* = 0.168. There were no statistically significant sociodemographic differences between completers and noncompleters at predeployment.

At pretraining, participants reported lifetime exposure to a mean of 5.78 (*SD* = 4.44) different PPTE types. At predeployment, participants reported exposure during the CTP to an average of 0.58 (*SD* = 2.31) different PPTE types. Table 1 presents exposure prevalence results for completers at pretraining and predeployment. See Supplementary Table S2 for the expanded version of Table 1. At pretraining, completers most frequently reported exposure (i.e., direct or indirect) to physical assault (*n* = 262, 58.4%), serious transport accidents (*n* = 229, 51.0%), and serious accidents at work home or during a recreational activity (*n* = 203, 45.2%). At predeployment, participants most frequently reported exposure (i.e., direct or indirect) to serious transport accidents (*n* = 27, 6.0%), physical assault (*n* = 26, 5.8%), and sudden accidental death (*n* = 23, 5.1%).

**TABLE 1:** Prevalence of potentially psychologically traumatic event (PPTE) exposure types for Royal Canadian Mounted Police Study completers

	Pretraining^b^		Predeployment^c^	
Exposure type^a^	*n* ^d^	%	*n* ^e^	%
No exposure	59	13.2	374	83.3
Life‐threatening natural disaster	184	41.0	17	3.8
Fire or explosion	192	42.8	21	4.7
Serious transportation accident	229	51.0	27	6.0
Serious accident at work, home, or during recreational activity	203	45.2	17	3.8
Exposure to a toxic substance	109	24.3	13	2.9
Physical assault	262	58.4	26	5.8
Assault with a weapon	161	35.9	18	4.0
Sexual assault	164	36.5	14	3.1
Other unwanted or uncomfortable sexual experience	183	40.8	16	3.6
Combat	48	10.7	6	1.3
Captivity	44	9.8	–^f^	–^f^
Life‐threatening illness or injury	189	42.1	18	4.0
Severe human suffering	134	29.8	9	2.0
Sudden violent death	185	41.2	20	4.5
Sudden accidental death	185	41.2	23	5.1
Serious injury, harm, or death you caused to someone else	59	13.1	7	1.6
Any other very stressful event or experience	53	11.8	5	1.1

^a^ Prevalence is based on the number of participants who reported they were “ever exposed” for each PPTE type divided by the total number of participants at predeployment (*n* = 449). For prevalence for each exposure modality (i.e., “happened to me,” “witnessed it,” “learned about it,” or “part of my public safety job”), see Supplementary Table S1.

^b^ Lifetime prevalence.

^c^ Prevalence during the CTP.

^d^ At pretraining, participants were not required to answer all questions; therefore, total percentages may not sum to 100.0% and *n* values may not sum to 449 due to nonresponse.

^e^ Based on responses to qualifying questions, not all participants at predeployment were presented with the Life Events Checklist for *DSM‐5*; therefore, total percentages may not sum to 100.0% and *n* values may not sum to 449.

^f^ Sample size was between *n* = 1 and *n* = 4, so data are not presented.

At pretraining, completers most frequently reported direct exposure (i.e., “it happened to me”) to physical assault (*n* = 138, 52.7%), other unwanted or uncomfortable sexual experience (*n =* 65, 35.5%), and any other very stressful event or experience (*n =* 25, 47.2%). At predeployment, completers most frequently reported direct exposure to physical assault (*n =* 13 of 26), other unwanted or uncomfortable sexual experience (*n =* 11 of 16), and serious transporation accident (*n =* 8 of 27). To protect the anonymity of participants (Information and Privacy Commissioner of Ontario, 2016), direct exposure prevalence (i.e., frequencies and percentages) across gender categories could not be presented due to small sample sizes across PPTE types. At predeployment, men most frequently reported direct exposure to physical assault and serious transportation accidents whereas women most frequently reported direct exposure to physical assault, other unwanted or uncomfortable sexual experience, and sexual assault.

At predeployment, the total number of different PPTE types was statistically significantly associated with increased odds of screening positive for any mental health disorder (i.e., PTSD, MDD, GAD, SAD, PD, or AUD), a*OR* = 1.22, 95% CI [1.01, 1.49], *p* = .049. We did not observe statistically significant associations between exposure to any PPTE type via any modality (i.e., any LEC‐5 exposure category) at predeployment and the odds of screening positive for any mental health disorder, a*OR* = 3.84, 95% CI [0.34, 42.99], *p* = .275. Post hoc sensitivity analyses did not indicate a deviation in model fit due to the inclusion of sociodemographic covariates and, thus, did not increase the risk of a Type II error.

Table 2 presents the linear regressions assessing associations between the total number of PPTE types endorsed and mental health disorder symptom scores at predeployment. At predeployment, the total number of PPTE types endorsed during the CTP was positively associated with self‐reported symptoms of PTSD, *B* = 1.12 (*SE* = 0.21), *F*(7, 256) = 4.761, *p* < .001; MDD, *B* = 0.43 (*SE* = 0.10), *F*(7, 351) = 3.986, *p* < .001; and GAD, *B =* 0.43 (*SE* = 0.13), *F*(7, 353) = 3.543, *p* <. 001, but not SAD, PD, or AUD, *p*s = .010–.358).

**TABLE 2:** Associations between mental disorder symptom scores and the total number of different potentially psychologically traumatic event (PPTE) types endorsed

Variable	Estimate^a^	*SE*	95% CI	*F*	*df*	*p*	*R* ^2^
Posttraumatic stress disorder	1.128	0.159	[0.816, 1.440]	7.104	8,388	< .001	.101
Major depressive disorder	0.383	0.084	[0.219, 0.548]	4.574	8,436	< .001	.045
Generalized anxiety disorder	0.364	0.099	[0.170, 0.557]	3.689	8,436	< .001	.029
Social anxiety disorder	0.266	0.103	[0.064, 0.467]	2.590	8,435	.010	.015
Panic disorder	0.750	0.395	[−0.162, 1.662]	1.897	8,25	.094	.008
Alcohol use disorder	0.064	0.070	[−0.073, 0.202]	0.920	8,357	.358	.002

*Note*: Models were adjusted for sociodemographic covariates, including sex, gender, age, level of education, ethnicity, marital status, and previous public safety experience. CI = confidence interval.

^a^ Unstandardized coefficient.

## DISCUSSION

The current study presents PPTE exposures among RCMP cadets from pretraining to predeployment and provides the first independent assessment of cadets’ PPTE exposures during the CTP. The results provide novel evidence of cadet PPTE exposures during the CTP and before deployment as active duty RCMP officers. Knowledge about the PPTEs that cadets experience during the CTP can inform recruitment and retention by providing insight into experiences that may impact cadets’ mental health, thereby informing resources needed to further support cadets during training. Information on PPTE exposures prior to deployment can inform mental health supports and resources needed to mitigate the impact of early career exposure.

At predeployment, most participants reported no critical incident exposures and, by extension, no PPTE exposures compared to those who reported exposure to one or more critical incidents during the CTP (*n* = 374, 83.3% vs. *n* = 75 16.7%). Lower exposure frequencies and exposure to fewer total PPTE types during the CTP were expected due to the difference in measurement periods (i.e., lifetime exposure vs. 6 months at the CTP). The limited vocational responsibilities and duties of cadets during training also likely impacted exposure prevalence. During the CTP, cadets spend more than 8 hr a day training in the classroom and on RCMP Depot grounds (RCMP, 2019) and are not deployed to respond to incidents or interact with the general public; nevertheless, cadets reported exposure to more PPTE types during the CTP than lifetime estimates for the Canadian general population (i.e., approximately two exposure types; Van Ameringen et al., 2008). The results further support the contention that RCMP members, even while in training, are exposed to a diverse range of PPTEs more frequently than the general population. Additionally, although PPTE exposure levels were lower during the CTP compared to over the lifetime, the prevalence of exposure during the CTP warrants action to improve the safety of cadets.

Among cadets who reported exposure to any PPTEs during the CTP, serious transport accident was reported most frequently, with 27 of 75 participants reporting exposure through any modality (i.e., direct or indirect). Some cadets also reported exposure to physical assault (*n =* 26) and sudden accidental death (*n =* 23) during the CTP. Most exposure to serious transport accidents and sudden accidental death was indirect (i.e., responses of “witnessed it,” “learned about it,” “part of my job”) and likely occurred during the CTP as part of standard training. The PPTE types reported at predeployment were similar to those most frequently reported by serving RCMP officers (Carleton et al., 2019), suggesting that PPTE exposure begins accumulating as early as recruit training.

RCMP officers and other public safety personnel are repeatedly exposed to PPTEs as a function of their work environments (Carleton et al., 2019; McFarlane, 2014). The accumulation of PPTE exposure in terms of both frequency and variety appears to be an important risk factor related to developing a posttraumatic stress injury, including PTSD (APA, 2013; Carleton et al., 2019). In the current study, the total number of different PPTE types endorsed was statistically significantly associated with increased odds of screening positive for any mental health disorder and with increased symptoms of PTSD, MDD, and GAD, but any exposure (i.e., exposure to at least one or more) was not, underscoring the impact of cumulative PPTE exposures. The associations observed between PPTE exposures and mental health are consistent with previous research and observations made at pretraining (Andrews et al., 2023; Carleton et al., 2019). However, the current results highlight how PPTE exposures can accumulate as early as training, increase the risk for trauma‐related symptoms and disorders, and warrant action to mitigate mental health challenges among cadets during training and onward throughout their careers. The current results should be interpreted cautiously because of how few cadets reported PPTE exposures at predeployment or screened positive for any mental health disorders.

Cadets reported the most frequent direct exposure (i.e., “it happened to me”) to physical assault, other unwanted or uncomfortable sexual experience, and serious transportation accident. During the CTP, cadets spend most of their time training and living on RCMP Depot grounds (i.e., the CTP campus). Evening and weekend activities are often required, leaving cadets with relatively little free time (RCMP, 2023). Cadets also do not typically interact with civilians or members of the public. Accordingly, PPTEs experienced during the CTP likely occur at the RCMP Depot, but participants were not asked to report detailed information to clarify the context (i.e., location, perpetrators) of any reported PPTE exposure. Notwithstanding, the results suggest that physical and other unwanted or uncomfortable sexual experience are noteworthy issues during CTP.

Gender differences were observed for the most frequent direct exposure types. Physical assault was reported equally by both men and women, whereas other unwanted or uncomfortable sexual experience was reported by mostly women, and direct exposure to sexual assault was reported by only women. The differences in the prevalence of sexual assault, other unwanted or uncomfortable sexual experiences, and physical assault observed in the current study are consistent with gender patterns observed in the Canadian general population (Cotter & Savage, 2019), among students attending Canadian Military College (Maxwell, 2020), and in Canadian postsecondary students (Burczycka, 2020). Approximately 15% of women attending Canadian Military College (Maxwell, 2020) and 11% of women attending Canadian postsecondary institutions (Burczycka, 2020) reported being sexually assaulted over a 12‐month period, which appears higher than men attending the same institutions (3.6% and 4%, respectively). Men in the general population tend to be more likely to be physically assaulted, whereas women are more likely to experience sexual assault or unwanted or uncomfortable sexual experiences (Burczycka, 2020; Cotter & Savage, 2019). Women in the general population are also more likely to experience unwanted sexual behavior in the workplace than men (29.0% vs. 17.0%; Cotter & Savage, 2019). Women police officers tend to be exposed to more sexualization, disrespect, sexually charged threats, and violence than men (Batton & Wright, 2019). Physical abuse and sexual misconduct in training environments have serious consequences for survivors, who often experience diminished feelings of trust for the organization; reductions in cohesion built during training; a lack of confidence; a fear of retribution, which prevents reporting; and decreased participation and concentration during training activities (Sweeting & Cole, 2023). Additionally, police who have experienced sexual misconduct perpetrated by a colleague have been reported to have lower productivity levels and higher levels of stress (Brown et al., 2018). Physical assault, sexual assault, and other unwanted or uncomfortable sexual experiences have also been associated with diverse mental health disorders among PSP (Carleton et al., 2019).

The current results indicate that additional training for RCMP employees working at the RCMP Depot should be implemented to increase the safety of cadets and identify and mitigate signs of physical abuse (e.g., bullying, nonsexual harassment behaviors) and sexual misconduct (i.e., unwanted or uncomfortable sexual experience and sexual assault) during the CTP. Additional training or communication for cadets may also help those who are exposed to a PPTE with accessing available supports, which may further aid in building and maintaining trust and safety within the RCMP. There may also be opportunities to further maximize the beneficial impact of available resources, such as the Independent Center for Harassment Resolution (RCMP, 2022), an independent unit that facilitates the resolution of harassment and violence for RCMP members, or applying similar prevention approaches used by the military (Farris et al., 2019; Potter & Stapleton, 2012).

The RCMP Study has several strengths and limitations (for details see Carleton et al., 2022). The strengths of the current paper include a large representative sample of cadets and a longitudinal design that allowed for assessments of PPTE exposure and mental health over the course of training to service deployment. The current study also has several limitations that can inform directions for future research. The voluntary nature of cadet participation in the RCMP Study creates an unknowable influence of self‐selection biases relative to cadets who chose to participate in the RCMP Study. The interruption of a sequential experimental cohort design due to the COVID‐19 pandemic led data collection to include participants from pre‐ and post‐COVID‐19 pandemic onset. Participants self‐reported symptoms related to various mental health disorders. A positive screen on a self‐report survey measure is not necessarily synonymous with meeting the diagnostic criteria for a given disorder, which requires a clinical interview by a licensed professional. The reduced number of participants who completed the PCL‐5 at predeployment due to a technical error is not expected to have artificially deflated the prevalence of positive PTSD screens among completers; only one participant who screened positive for PTSD at pretraining, and who explicitly reported no critical incidents during the CTP, did not receive the PCL‐5 at predeployment. Additionally, the small number of positive mental health disorder screens at predeployment was observed for all measures of mental health disorders, not just the PCL‐5. The current results assessed the frequency of exposure to diverse critical incidents and PPTEs using retrospective recall. Future research should consider assessing PPTE exposure by using data from the extant multimodal tools in the RCMP Study (e.g., daily and monthly surveys, structured clinical interviews) and expanding the breadth of data collection with such tools. The large sample size, diverse assessment modalities with clearly repeated survey instructions, analyses, paid time to complete RCMP Study tasks, preregistration of hypotheses, third‐party assessors, and participant anonymity may offset the overall and current limitations.

Technical errors in the survey limited the reporting and subsequent analyses of critical incident endorsement and PPTE exposure at predeployment. The critical incidents most affected by such technical errors were response options for “experienced or witnessed sexual assault/sexual harassment/sexual discrimination,” and “experienced or witnessed any other potentially psychologically traumatic event.” The two critical incident response options were added to predeployment surveys during data collection, meaning that a subset of participants (*n =* 278) was not presented with either and selected “none” in response to the list of available critical incidents, subsequently skipping the administration of the LEC‐5. This technical error may have resulted in the current results underrepresenting PPTE exposure among participants because only some participants (*n =* 75) received the LEC‐5 based on their responses to the critical incident question. The current results may also underrepresent the true prevalence of PPTEs due to participants responding “prefer not to answer” and because individuals who experienced a PPTE during the CTP may have left the RCMP Study or the CTP. Additionally, the current results may underrepresent the true prevalence of sexual assault and other unwanted or uncomfortable sexual experience, as underreporting of sexual victimization is common (Cotter & Savage, 2019). Although the low prevalence of mental health disorders and PPTEs in the current study may be due in part to the technical errors in the survey, they may also be due to underreporting. Underreporting of traumatic events is common due to issues with retrospective recall when using the LEC‐5 (Krinsley et al., 2003), whereas the underreporting of mental health disorder symptoms is common due to perceived stigma or worries about discrimination in the workplace (Marshall et al., 2021; Rona et al., 2017). As the current data were collected in the context of the training environment, it is possible that perceived stigma and worries about discrimination or reprisals led to the underreporting of PPTE exposure. Nonetheless, the current data accurately indicate that PPTE exposure occurs during the CTP and highlight the need to further increase RCMP cadet safety during the CTP.

In addition, due to technical errors, PPTE exposure frequency (i.e., the number of times a participant experienced each exposure type) and indications as to which PPTE participants perceived as their worst PPTE were not available for inclusion in the current paper. The technical errors have been corrected, which will support a more robust assessment of PPTE exposures during the CTP data collection is completed with participants in “augmented” training condition as a part of the larger RCMP Study.

The current results offer empirical evidence of PPTE exposures reported by RCMP cadets from pretraining to predeployment, which encompasses the 26‐week CTP. The results indicate that during the CTP, 16.7% of cadets reported PPTE exposures. The most frequently reported PPTEs were typically experienced indirectly (e.g., responses of “witnessed it,” “learned about it,” or “part of my job”). The current results further support possible associations between cumulative PPTE exposure and mental health among police officers, underscoring the need for early and ongoing evidence‐based assessments, support, and interventions. The direct exposure reported by cadets during the CTP provides evidence of opportunities to further increase RCMP cadet safety during the CTP. The results also indicate that additional research is needed to understand the contexts of PPTE exposure during the CTP, particularly to clarify who was involved, where the PPTE occurred, what could have been done to prevent the PPTE, and what was done to mitigate the impact of the PPTE. Nevertheless, the current data indicate that additional practices and procedures are needed to try and prevent PPTE exposure that impacts the health and safety of cadets. In the interim, additional training or communication for RCMP employees may help to mitigate behaviors that facilitate PPTEs as well as to identify and deal with perpetrators of unsafe or harmful behaviors. Additional communication is also needed to help cadets understand what resources are available to help in the event of a PPTE during the CTP.

## AUTHOR NOTE

The Royal Canadian Mounted Police (RCMP) Study is supported by the RCMP, the Government of Canada, and the Ministry of Public Safety and Emergency Preparedness. Lisa M. Lix is supported by a Tier I Canada Research Chair in Methods for Electronic Health Data Quality. Tracie O. Afifi is supported by a Tier I Canada Research Chair in Childhood Adversity and Resilience. Gordon J.G. Asmundson is supported by a University of Regina President's Research Chair. The development, analyses, and distribution of the current article was supported by a generous grant from the Medavie Foundation.

The RCMP Study is made possible by a large and diverse team, with detailed acknowledgments available online (www.rcmpstudy.ca) and in the study protocol paper. Correspondence regarding the described study should be addressed to rcmpstudy@uregina.ca. Additional information is available in English and French at www.rcmpstudy.ca

## OPEN PRACTICES STATEMENT

The RCMP Study hypotheses were preregistered. Hypotheses specific to individual difference variables are provided in the Supplemental Tables (see http://hdl.handle.net/10294/14680).

The datasets presented in this article are not readily available due to the sensitive nature of the content and to protect the anonymity of participants. Requests to access the datasets should be directed to katie.andrews@uregina.ca.

## Supporting information



Supplementary Table S1. Mean total number of different types of potentially psychologically traumatic event (PPTE) exposures across sociodemographic categories for completers at pre‐training and pre‐deployment

Supplementary Table S2. Expanded Table of Prevalence of Potentially Psychologically Traumatic Event Exposure Types for Completers (n=449)
