# Supplementary material for: Royal Canadian Mounted Police cadets’ exposure to potentially psychologically traumatic events during the Cadet Training Program
Source: J Trauma Stress. 2024 Dec 20;38(2):234–46. doi: 10.1002/jts.23115 (PMC11967305; doi:10.1002/jts.23115)
Supplement: Supplementary file 1 — Supplementary Table S1. Mean total number of different types of potentially psychologically traumatic event (PPTE) exposures across sociodemographic categories for completers at pre‐training and pre‐deployment [file JTS-38-234-s002.docx]

Supplementary Table 1.

*Mean total number of different types of potentially psychologically traumatic event (PPTE) exposures across sociodemographic categories for completers at pre-training and pre-deployment*

|  | Demographic Distribution | | Total number of Different PPTE Types | | | | | | | | | |
| --- | --- | --- | --- | --- | --- | --- | --- | --- | --- | --- | --- | --- |
|  |  |  | Pre-Training | | | | | Pre-deployment | | | | |
|  | % | *n* | *Mean* | *SD* | *n^1^* | Test Statistic^3^ | Effect Size | *Mean* | *SD* | *n^2^* | Test Statistic^3^ | Effect Size |
| **Total Sample** |  |  |  |  |  |  |  |  |  |  |  |  |
| Total Sample | 100 | 449 | 5.78 | 4.44 | 447 |  |  | 0.58 | 2.31 | 449 |  |  |
| **Sex** |  |  |  |  |  |  |  |  |  |  |  |  |
| Male | 73.9 | 332 | 5.70 | 4.46 | 330 | *t*(443)=0.27 | .030 | 0.52 | 2.22 | 332 | *t*(443)=0.75 | .081 |
| Female | 25.4 | 114 | 5.83 | 4.29 | 114 |  |  | 0.71 | 2.65 | 114 |  |  |
| **Gender** |  |  |  |  |  |  |  |  |  |  |  |  |
| Man | 74.2 | 333 | 5.76 | 4.49 | 331 | *t*(440)=0.02 | .004 | 0.52 | 2.21 | 333 | *t*(440)=0.83 | .002 |
| Woman | 24.7 | 111 | 5.77 | 4.30 | 111 |  |  | 0.73 | 2.58 | 111 |  |  |
| Gender diverse^4^ | ^ | ^ | ^ |  | ^ |  |  | ^ | ^ | ^ |  |  |
| **Age** |  |  |  |  |  |  |  |  |  |  |  |  |
| 19-29 | 62.8 | 282 | 5.53 | 4.40 | 281 | *F*(2,428)=1.49 | .010 | 0.57 | 2.30 | 282 | *F*(2,430)=0.07 | .000 |
| 30-39 | 29.0 | 130 | 6.34 | 4.47 | 130 |  |  | 0.58 | 2.37 | 130 |  |  |
| 40-49 | 4.7 | 21 | 5.80 | 4.42 | 20 |  |  | 0.38 | 1.75 | 21 |  |  |
| 50-59 | ^ | ^ | ^ |  | ^ |  |  | ^ | ^ | ^ |  |  |
| 60 and older | - | - | - |  | - |  |  | - | - | - |  |  |
| **Ethnicity** |  |  |  |  |  |  |  |  |  |  |  |  |
| Asian | 6.0 | 27 | 6.12 | 5.54 | 26 | *F*(5,414)=0.54 | .006 | 1.22 | 4.05 | 27 | *F*(5,416)=0.86 | .010 |
| Black | 1.3 | 6 | 6.67 | 5.43 | 6 |  |  | 0 | 0 | 6 |  |  |
| First Nations/Inuit/Metis | 2.2 | 10 | 3.70 | 4.30 | 10 |  |  | 0 | 0 | 10 |  |  |
| Hispanic | 1.3 | 6 | 6.17 | 5.15 | 6 |  |  | 0 | 0 | 6 |  |  |
| South Asian | 6.0 | 27 | 5.48 | 5.69 | 27 |  |  | 0.67 | 3.27 | 27 |  |  |
| White | 77.1 | 346 | 5.71 | 4.20 | 345 |  |  | 0.49 | 1.90 | 346 |  |  |
| **Marital Status** |  |  |  |  |  |  |  |  |  |  |  |  |
| Single | 45.9 | 206 | 5.53 | 4.52 | 206 | *F*(2,412)=1.17 | .006 | 0.89 | 3.00 | 206 | *F*(2,413)=3.81 | .018 |
| Separated/Divorced/Widowed | 1.1 | 5 | 4.80 | 3.11 | 5 |  |  | 0 | 0 | 5 |  |  |
| Married/Common-Law | 45.7 | 205 | 6.16 | 4.39 | 204 |  |  | 0.27 | 1.38 | 205 |  |  |
| **Province of Residence^4^** | | | | | | | | | | | | |
| Western Canada | 53.9 | 242 | 6.17 | 4.57 | 241 | *F*(2,438)=2.55 | .012 | 0.70 | 2.63 | 242 | *F*(2,440)=2.01 | .010 |
| Eastern Canada | 32.1 | 144 | 5.40 | 4.43 | 143 |  |  | 0.59 | 2.16 | 144 |  |  |
| Atlantic Canada | 12.7 | 57 | 4.91 | 3.81 | 57 |  |  | 0.02 | 0.13 | 57 |  |  |
| Northern Territories | ^ | ^ | ^ |  | ^ |  |  | ^ | ^ | ^ |  |  |
| **Education** |  |  |  |  |  |  |  |  |  |  |  |  |
| High school graduate or less | 9.8 | 44 | 5.60 | 4.44 | 43 | *F*(2,424)=2.26 | .011 | 0.20 | 1.36 | 44 | *F*(2,426)=1.62 | .007 |
| Some post-secondary school | 43.9 | 197 | 6.31 | 4.51 | 197 |  |  | 0.81 | 2.66 | 197 |  |  |
| University degree/4-year college or higher | 41.9 | 188 | 5.35 | 4.45 | 187 |  |  | 0.49 | 2.20 | 188 |  |  |
| **Previous PSP or Military Experience** | | | | | | | | | | | | |
| Yes | 30.1 | 135 | 7.35 | 4.54 | 133 | *t*(406)=4.90 | 0.516*** | 0.48 | 2.13 | 135 | *t*(409)=1.47 | 0.168 |
| No | 61.2 | 275 | 5.08 | 4.31 | 275 |  |  | 0.87 | 2.69 | 275 |  |  |

Notes: ^***^*p* < .001 – Statistically significantly different. ^1^ Participants at T1 were not required to answer all questions, therefore total percentages may not sum to 100% and frequencies (*n*) may not sum to 449 due to non-response. ^2^ Based on responses to qualifying questions, not all participants at T2 were presented the Life Events Checklist for the DSM-5, therefore total percentages may not sum to 100% and ns may not sum to 449. ^3^ The test results comparing scores on mental disorder screening measures across categorical participant demographics; *t*(degrees of freedom)=test statistic; *F*(numerator degrees of freedom, denominator degrees of freedom)=test statistic. ^4^ Gender Diverse includes Non-binary, Transgender, and Two-spirt categories. ^4^ Western Canada = Alberta, British Columbia, Manitoba, Saskatchewan; Eastern Canada = Ontario, Quebec; Atlantic Canada = New Brunswick, Newfoundland and Labrador, Nova Scotia, Prince Edward Island; Northern Territories = Nunavut, Northwest Territories, Yukon. - = *n* = 0; ^ = Sample size between 1 and 4, so data not presented.
