# Supplementary material for: Royal Canadian Mounted Police cadets’ exposure to potentially psychologically traumatic events during the Cadet Training Program
Source: J Trauma Stress. 2024 Dec 20;38(2):234–46. doi: 10.1002/jts.23115 (PMC11967305; doi:10.1002/jts.23115)
Supplement: Supplementary file 2 — Supplementary Table S2. Expanded Table of Prevalence of Potentially Psychologically Traumatic Event Exposure Types for Completers (n=449) [file JTS-38-234-s001.docx]

Supplementary Table S2.

*Expanded Table of Prevalence of Potentially Psychologically Traumatic Event Exposure Types for Completers (n=449)*

| Type of Exposure | Pre-Training  (Lifetime Prevalence) | | Pre-deployment  (Prevalence during the CTP) | |
| --- | --- | --- | --- | --- |
|  | % | *n*^1^ | % | *n*^2^ |
| No Exposures | 13.2 | 59 | 83.3 | 374 |
| Life threatening natural disaster |  |  |  |  |
| Ever exposed ^3, 4^ | 41.0 | 184 | 3.8 | 17 |
| Happened to me ^5^ | 28.8 | 53 | 35.3 | 6 |
| Witnessed it | 23.4 | 43 | 35.3 | 6 |
| Learned about it | 51.6 | 95 | 35.3 | 6 |
| Part of my public safety job | 13.6 | 25 | ^ | ^ |
| Fire or explosion |  |  |  |  |
| Ever exposed | 42.8 | 192 | 4.7 | 21 |
| Happened to me | 14.1 | 27 | ^ | ^ |
| Witnessed it | 33.3 | 64 | 28.6 | 6 |
| Learned about it | 47.9 | 92 | 23.8 | 5 |
| Part of my public safety job | 23.4 | 45 | 47.6 | 10 |
| Serious transportation accident |  |  |  |  |
| Ever exposed | 51.0 | 229 | 6.0 | 27 |
| Happened to me | 24.9 | 57 | 29.6 | 8 |
| Witnessed it | 29.7 | 68 | 37.0 | 10 |
| Learned about it | 48.0 | 110 | 33.3 | 9 |
| Part of my public safety job | 16.6 | 38 | 33.3 | 9 |
| Serious accident at work, home, or during recreational activity | | | | |
| Ever exposed | 45.2 | 203 | 3.8 | 17 |
| Happened to me | 24.6 | 50 | 29.4 | 5 |
| Witnessed it | 38.4 | 78 | 41.2 | 7 |
| Learned about it | 47.3 | 96 | 41.2 | 7 |
| Part of my public safety job | 17.2 | 35 | 29.4 | 5 |
| Exposure to toxic substance |  |  |  |  |
| Ever exposed | 24.3 | 109 | 2.9 | 13 |
| Happened to me | 19.3 | 21 | ^ | ^ |
| Witnessed it | 16.5 | 18 | - | - |
| Learned about it | 56.9 | 62 | ^ | ^ |
| Part of my public safety job | 28.4 | 31 | 69.2 | 9 |
| Physical assault |  |  |  |  |
| Ever exposed | 58.4 | 262 | 5.8 | 26 |
| Happened to me | 52.7 | 138 | 50.0 | 13 |
| Witnessed it | 43.1 | 113 | 30.8 | 8 |
| Learned about it | 37.0 | 97 | 38.5 | 10 |
| Part of my public safety job | 22.1 | 58 | 42.3 | 11 |
| Assault with a weapon |  |  |  |  |
| Ever exposed | 35.9 | 161 | 4.0 | 18 |
| Happened to me | 21.1 | 34 | ^ | ^ |
| Witnessed it | 25.5 | 41 | ^ | ^ |
| Learned about it | 55.3 | 89 | 44.4 | 8 |
| Part of my public safety job | 26.1 | 42 | 38.9 | 7 |
| Sexual assault |  |  |  |  |
| Ever exposed | 36.5 | 164 | 3.1 | 14 |
| Happened to me | 16.5 | 27 | 35.7 | 5 |
| Witnessed it | 4.9 | 8 | ^ | ^ |
| Learned about it | 76.8 | 126 | 35.7 | 5 |
| Part of my public safety job | 14.6 | 24 | ^ | ^ |
| Other unwanted or uncomfortable sexual experience |  |  |  |  |
| Ever exposed | 40.8 | 183 | 3.6 | 16 |
| Happened to me | 35.5 | 65 | 68.8 | 11 |
| Witnessed it | 15.8 | 29 | ^ | ^ |
| Learned about it | 62.3 | 114 | ^ | ^ |
| Part of my public safety job | 12.6 | 23 | ^ | ^ |
| Combat |  |  |  |  |
| Ever exposed | 10.7 | 48 | 1.3 | 6 |
| Happened to me | 12.5 | 6 | - | - |
| Witnessed it | ^ | ^ | - | - |
| Learned about it | 85.4 | 41 | 83.3 | 5 |
| Part of my public safety job | 12.5 | 6 | ^ | ^ |
| Captivity |  |  |  |  |
| Ever exposed | 9.8 | 44 | ^ | ^ |
| Happened to me | - | - | - | - |
| Witnessed it | ^ | ^ | - | - |
| Learned about it | 84.1 | 37 | ^ | ^ |
| Part of my public safety job | 20.5 | 9 | ^ | ^ |
| Life threatening illness or injury |  |  |  |  |
| Ever exposed | 42.1 | 189 | 4.0 | 18 |
| Happened to me | 11.1 | 21 | ^ | ^ |
| Witnessed it | 56.6 | 107 | 50.0 | 9 |
| Learned about it | 53.4 | 101 | 38.9 | 7 |
| Part of my public safety job | 15.9 | 30 | 33.3 | 6 |
| Severe human suffering |  |  |  |  |
| Ever exposed | 29.8 | 134 | 2.0 | 9 |
| Happened to me | ^ | ^ | - | - |
| Witnessed it | 47.0 | 63 | ^ | ^ |
| Learned about it | 55.2 | 74 | ^ | ^ |
| Part of my public safety job | 20.9 | 28 | 55.6 | 5 |
| Sudden violent death |  |  |  |  |
| Ever exposed | 41.2 | 185 | 4.5 | 20 |
| Happened to me | ^ | ^ | ^ | ^ |
| Witnessed it | 21.6 | 40 | 25.0 | 5 |
| Learned about it | 72.4 | 134 | 50.0 | 10 |
| Part of my public safety job | 20.5 | 38 | 45.0 | 9 |
| Sudden accidental death |  |  |  |  |
| Ever exposed | 41.2 | 185 | 5.1 | 23 |
| Happened to me | 5.9 | 11 | - | - |
| Witnessed it | 25.9 | 48 | 34.8 | 8 |
| Learned about it | 66.5 | 123 | 43.5 | 10 |
| Part of my public safety job | 21.1 | 39 | 39.1 | 9 |
| Serious injury, harm, or death you caused to someone else | | | | |
| Ever exposed | 13.1 | 59 | 1.6 | 7 |
| Happened to me | 8.5 | 5 | ^ | ^ |
| Witnessed it | 23.7 | 14 | ^ | ^ |
| Learned about it | 71.2 | 42 | ^ | ^ |
| Part of my public safety job | 27.1 | 16 | ^ | ^ |
| Any other very stressful event or experience |  |  |  |  |
| Ever exposed | 11.8 | 53 | 1.1 | 5 |
| Happened to me | 47.2 | 25 | ^ | ^ |
| Witnessed it | 34.0 | 18 | ^ | ^ |
| Learned about it | 24.5 | 13 | ^ | ^ |
| Part of my public safety job | 26.4 | 14 | ^ | ^ |

*Note*. ^1^ Participants at pre-training were not required to answer all questions, therefore total percentages may not sum to 100% and *n*s may not sum to 449 due to non-response. ^2^ Based on responses to qualifying questions, not all participants at pre-deployment were presented the Life Events Checklist for the DSM-5, therefore total percentages may not sum to 100% and ns may not sum to 449. ^3^ Responses for Happened to me, Witnessed it, Learned about it, and Part of my public safety job were not mutually exclusive therefore responses are based on the total number of participants reporting ever exposed (i.e. exposure through any modality) for each PPTE type. ^4^ Percentages for Ever Exposed are based on the number of participants reporting ever exposed for each PPTE type divided by the total number of participants at T2 (n=449). ^5^ Percentages for responses Happened to me, Witnessed it, Learned about it, and Part of my public safety job were calculated based on the number of participants reporting each modality of exposure divided by the number of participants who reporting ever exposed for each PPTE type.

- = *n* = 0; ^ = Sample size between 1 and 4, so data not presented
